# Supplementary material for: Comparative study of SPI success factors in global and in-house environment for large-scale software companies
Source: PeerJ Comput Sci. 2023 Nov 20;9:e1656. doi: 10.7717/peerj-cs.1656 (PMC10703003; doi:10.7717/peerj-cs.1656)
Supplement: Supplemental Information 1 [file peerj-cs-09-1656-s001.pdf]

Comparative Study of SPI SuccessFactors in Global and In-house Environment for Large Scale Software Companies

I am a student of MS Software Engineering at COMSATS University Islamabad and conducting research for my MS thesis. From the literature, we have identified Software process improvement (SPI) success factors, for large scale software development companies, in case of Global Software development (GSD) and In-house softwaredevelopment. The list of success factors in each case is given below. Please sparesome time to add additional success factors (if any) to the lists.

javedkhushi2@gmail.com Switch account

* Required

Email *

Your email

Position/Job title: *

Your answer

Experience in years *

Your answer

Company's country in which it is located: *

Your answer

What is primary business function of your company (You can select both as well) *

- [ ] Inhouse software development
- [ ] Global software development

Request edit access

Section-II

Through the systematic literature review, we have identified the following 35 SPI success factors in GSD context, for large scale software development companies. Please list the additional success factors that you think are important for SPI in GSD context (if any).

IDs	Success factors	IDs	Success factors
G_1	SPI leadership	G_{21}	Joint management infrastructure
G_2	Efficient project management	G_{22}	SPI consultancy
G_3	Track record of successful projects	G_{23}	Staff involvement
G_4	Political stability	G_{24}	Organizational culture
G_5	Pilot project performance	G_{25}	Information Sharing
G_6	Data protection laws	G_{26}	Senior management commitment and support
G_7	SPI certification laws	G_{27}	Organizational infrastructure
G_8	Efficient contract management	G_{28}	Overseas site response
G_9	Knowledge of clients' languageand culture	G_{29}	SPI expertise
G_{10}	Continuous organizational support	G_{30}	Effective requirement analysis
G_{11}	SPI standards and procedures	G_{31}	Effective customer involvement
G_{12}	Mutual understanding among members	G_{32}	Motivation
G_{13}	Process improvement evaluation	G_{33}	Training
G_{14}	3Cs (control, communication, coordination)	G_{34}	Risk sharing
G_{15}	Skilled human resources	G_{35}	Rich technology infrastructure
G_{16}	Setting SPI goals		
G_{17}	Reward schemes		
G_{18}	Allocation of resources		
G_{19}	Trust		
G_{20}	SPI awareness		

Your answer

Section-III

Through the systematic literature review, we have identified the following 33 SPI success factors in In-house context, for large scale software development companies. Please list the additional success factors that you think are important for SPI in In-house context (if any).

IDs	Success factors	IDs	Success factors
I_1	Senior management commitment and support	I_{20}	Customer Involvement
I_2	Staff involvement	I_{21}	Skills
I_3	Experience of staff	I_{22}	Time allocation
I_4	Training	I_{23}	Motivation
I_5	Allocation of resources	I_{24}	Project managers personality
I_6	Communication	I_{25}	Team leader support
I_7	SPI goals	I_{26}	Organizational structure
I_8	Tools	I_{27}	SPI consultancy
I_9	Reward schemes	I_{28}	Client support
I_{10}	Monitoring and feedback	I_{29}	Employee support
I_{11}	SPI leadership and procedures	I_{30}	Participation of top leader
I_{12}	Teamwork	I_{31}	Automated tools
I_{13}	Change management	I_{32}	Tailoring of process
I_{14}	Roles and responsibilities	I_{33}	Managing the project
I_{15}	SPI personal respect		
I_{16}	Exploitation of existing knowledge		
I_{17}	SPI awareness		
I_{18}	Training and mentoring		
I_{19}	Exploration of new knowledge		

Your answer

Submit

Clear form

Never submit passwords through Google Forms.

This content is neither created nor endorsed by Google. Report Abuse - Terms of Service - Privacy Policy

Google Forms
