# Supplementary material for: Comparative study of SPI success factors in global and in-house environment for large-scale software companies
Source: PeerJ Comput Sci. 2023 Nov 20;9:e1656. doi: 10.7717/peerj-cs.1656 (PMC10703003; doi:10.7717/peerj-cs.1656)
Supplement: Supplemental Information 2 [file peerj-cs-09-1656-s002.pdf]

Comparative Study of SPI SuccessFactors in Global and In-house Environment for Large Scale Software Companies

I am a student of MS Software Engineering at COMSATS University Islamabad and conducting research for my MS thesis. For the large scale software development companies, we have identified common success factors of Software process improvement (SPI)* in case of Global Software development (GSD)** and In-house*** software development from the literature and survey. The success factors are given below along with Likert scale. Please spare some time to rate the SPI success factorsaccording to their importance in two different scenarios. Please do compare the successfactor that is more important in GSD or in In-house

*Software Process Improvement methodology is defined as a sequence of tasks, tools, and techniques to plan and implement improvement activities to achieve specific goals such as increasing development speed, achieving higher product quality or reducing costs.
**When the distribution of the members of a distributed software development teamexceeds the frontiers of a country.
***In-house refers to an activity or operation that is performed within a company, instead of relying on outsourcing. The firm uses its own employees and resources to develop a software.

javedkhushi2@gmail.com (not shared) Switch account

* Required

Email *

Your answer

Position/Job title: *

Your answer

Experience in years *

Your answer

Company's country in which it is located: *

Your answer

What is primary business function of your company (You can select both as well) *

- [ ] GSD
- [ ] Inhouse

Section-II

SPI Leadership

Request edit access

SPI Leadership in GSD *

	1	2	3	4	5	
less important	○	○	○	○	○	more important

SPI Leadership in inhouse *

	1	2	3	4	5	
less important	○	○	○	○	○	more important

Project management

Project management in GSD *

	1	2	3	4	5	
less important	○	○	○	○	○	more important

Project management in inhouse *

	1	2	3	4	5	
less important	○	○	○	○	○	more important

Communication

Communication in GSD *

	1	2	3	4	5	
less important	○	○	○	○	○	more important

Communication in inhouse *

	1	2	3	4	5	
less important	○	○	○	○	○	more important

Teamwork

Teamwork in GSD *

	1	2	3	4	5	
less important	○	○	○	○	○	more important

Request edit access

Teamwork in inhouse *

	1	2	3	4	5	
less important	○	○	○	○	○	more important

Setting SPI goal

Setting SPI goal in GSD *

	1	2	3	4	5	
less important	○	○	○	○	○	more important

Setting SPI goal in inhouse *

	1	2	3	4	5	
less important	○	○	○	○	○	more important

SPI awareness

SPI awareness in GSD *

	1	2	3	4	5	
less important	○	○	○	○	○	more important

SPI awareness in inhouse *

	1	2	3	4	5	
less important	○	○	○	○	○	more important

Allocation of resources

Allocation of resources in GSD *

	1	2	3	4	5	
less important	○	○	○	○	○	more important

Allocation of resources in inhouse *

	1	2	3	4	5	
less important	○	○	○	○	○	more important

Request edit access

SPI consultancy

SPI consultancy in GSD *

	1	2	3	4	5	
less important	◯	◯	◯	◯	◯	more important

SPI consultancy in inhouse *

	1	2	3	4	5	
less important	◯	◯	◯	◯	◯	more important

Staff involvement

Staff involvement in GSD *

	1	2	3	4	5	
less important	◯	◯	◯	◯	◯	more important

Staff involvement in inhouse *

	1	2	3	4	5	
less important	◯	◯	◯	◯	◯	more important

Senior management commitment and support

Senior management commitment and support in GSD *

	1	2	3	4	5	
less important	◯	◯	◯	◯	◯	more important

Senior management commitment and support in inhouse *

	1	2	3	4	5	
less important	◯	◯	◯	◯	◯	more important

Organizational infrastructure

Request edit access

Organizational infrastructure in GSD *

	1	2	3	4	5	
less important	○	○	○	○	○	more important

Organizational infrastructure in inhouse *

	1	2	3	4	5	
less important	○	○	○	○	○	more important

Customer involvement/ client support

Customer involvement/ client support in GSD *

	1	2	3	4	5	
less important	○	○	○	○	○	more important

Customer involvement/ client support in inhouse *

	1	2	3	4	5	
less important	○	○	○	○	○	more important

Motivation

Motivation in GSD *

	1	2	3	4	5	
less important	○	○	○	○	○	more important

Motivation in inhouse *

	1	2	3	4	5	
less important	○	○	○	○	○	more important

Training

Training in GSD *

	1	2	3	4	5	
less important	○	○	○	○	○	more important

Request edit access

Training in inhouse *

	1	2	3	4	5	
less important	◯	◯	◯	◯	◯	more important

Reward Schemes

Reward Schemes in GSD *

	1	2	3	4	5	
less important	◯	◯	◯	◯	◯	more important

Reward Schemes in inhouse *

	1	2	3	4	5	
less important	◯	◯	◯	◯	◯	more important

On time delivery

On time delivery in GSD *

	1	2	3	4	5	
less important	◯	◯	◯	◯	◯	more important

On time delivery in inhouse *

	1	2	3	4	5	
less important	◯	◯	◯	◯	◯	more important

Easy to understand documentation.

Easy to understand documentation in GSD *

	1	2	3	4	5	
less important	◯	◯	◯	◯	◯	more important

Easy to understand documentation in inhouse *

	1	2	3	4	5	
less important	◯	◯	◯	◯	◯	more important

Request edit access

Submit Clear form

Never submit passwords through Google Forms.

This content is neither created nor endorsed by Google. Report Abuse - Terms of Service - Privacy Policy

Google Forms

Request edit access
